# Personal

**Published:** 1904-07

**Authors:** 


### PERSONAL.

---

Dr. Charles G. Stockton, of Buffalo, at the recent meeting of the American Medical Association was chosen to deliver the oration in medicine at the Portland, Ore., session next June. Dr. Stockton, who is professor of medicine at the University of Buffalo, occupies an enviable position as an author and teacher, and the honor that has thus come to him is fittingly bestowed. He will adorn the place to which he has been elected, and we venture the opinion that his oration will be an important feature of the meeting.

---

Dr. Roswell Park, of Buffalo, delivered the address on surgery at the annual meeting of the State Medical Society of Wisconsin, at Milwaukee, June 23, 1904, choosing for his subject, The surgical treatment of "dyspepsia." We take pleasure in presenting this important paper in full elsewhere in this edition of the Journal.

---

Professor Dr. Albert Hoffa, of Berlin, the most eminent orthopedist in Germany, visited Buffalo during June, accompanied by his assistant, Dr. Spitze. During his stay in Buffalo, Dr. Hoffa was the guest of Dr. Roswell Park.

Dr. Ernest Wende, of Buffalo, was elected to membership in the American Dermatological Association, during its recent meeting held at Niagara Falls.

---

Dr. George Ben Johnston, of Richmond, was elected president of the American Surgical Association during its recent meeting at Saint Louis. Dr. Johnston is an exponent of the best surgery of the day, and is the leading surgeon south of the Potomac, therefore his elevation to the highest surgical honor in America is opportune, while he is yet in the most useful period of his life.

---

Dr. F. Park Lewis, of Buffalo, was elected president of the board of managers of the State School for the Blind, at Batavia, at the annual meeting of the board, held at the institute, June 1, 1904.

---

Dr. Frederick J. Barrett, of Buffalo, is spending the summer in Europe, taking a special course in ophthalmology, and visiting the eye clinics in the principal hospitals of London, Paris and Berlin.

---

Dr. Cyrus P. Peckham, surgeon United States Marine Hospital and Public Health Service, has been assigned to duty in Buffalo, vice Eugene Wasdin, transferred to Memphis.

---

Dr. Charles L. Bonifield, of Cincinnati, was elected chairman of the section of obstetrics and gynecology at the recent meeting of the American Medical Assciation. Dr. Bonifield's excellent service as secretary during several years past receives through this promotion most appreciative expression. Dr. Walter P. Manton, of Detroit, who was chosen secretary of the section, vice Dr. Bonifield promoted, is experienced in medical society affairs, and will prove a most capable officer.

---

Dr. E. C. Dudley, of Chicago, the distinguished teacher and the author of that superb textbook, "Dudley's Gynecology," was elected president of the American Gynecological Society during its recent meeting at Boston.

---

Dr. Lucien Howe, of Buffalo, sails in July to attend the International Ophthalmological Congress at Lucerne. He will read a paper, with demonstration, on "Photographic measurements of the time required for lateral movements of the eye." He also will take part in the meeting of the British Medical Association, and will stay with the ophthalmologic section at Keble College, Oxford.

